# Shift and Metabolic Potentials of Microbial Eukaryotic Communities Across the Full Depths of the Mariana Trench

**DOI:** 10.3389/fmicb.2020.603692

**Published:** 2021-01-18

**Authors:** Xiao-Yu Zhu, Jiwen Liu, Chun-Xu Xue, Jiwei Tian, Xiao-Hua Zhang

**Affiliations:** ^1^College of Marine Life Sciences, Ocean University of China, Qingdao, China; ^2^Laboratory for Marine Ecology and Environmental Science, Qingdao National Laboratory for Marine Science and Technology, Qingdao, China; ^3^Institute of Evolution & Marine Biodiversity, Ocean University of China, Qingdao, China; ^4^Frontiers Science Center for Deep Ocean Multispheres and Earth System, Ocean University of China, Qingdao, China

**Keywords:** Mariana Trench, microbial eukaryotes, metagenomics, metabolic potentials, diversity

## Abstract

Microbial eukaryotes are widespread and play important roles in marine ecosystems. However, their ecological characteristics in the deep sea (>1,000 m), especially hadal trenches, were largely unknown. Here, we investigated the diversity and metabolic potentials of microbial eukaryotes along the whole water column of the Mariana Trench by metagenomics. Our results showed clear depth-related distribution of microbial eukaryotic community and associated metabolic potentials. Surface seawater was dominated by phototrophic/mixotrophic groups (e.g., Dinoflagellata) and genes involved in biosynthesis (photosynthesis and fatty acid biosynthesis), while deep (bathypelagic and/or hadal) seawaters were enriched with heterotrophic groups (e.g., Bicoecea) and genes related to digestion (lysosomal enzymes and V-type ATPase) and carbohydrate metabolism. Co-occurrence analysis revealed high intra-domain connectivity, indicating that microbial eukaryotic composition was more influenced by microbial eukaryotes themselves than bacteria. Increased abundance of genes associated with unsaturated fatty acid biosynthesis likely plays a role in resisting high hydrostatic pressure. *Top1* and *hupB* genes, responsible for the formation and stabilization of DNA structure, were unique and abundant in the hadal zone and thus may be helpful to stabilize DNA structure in the deep sea. Overall, our results provide insights into the distribution and potential adaptability of microbial eukaryotes in the hadal zone.

## Introduction

With high morphological and genetic variability, microbial eukaryotes fulfill a variety of crucial ecological roles and are essential participants of marine carbon cycle (Azaml et al., 1983; Worden et al., 2015). Phototrophic microbial eukaryotes are important contributors to global primary productivity (Field et al., 1998; De Vargas et al., 2015), while heterotrophic microbial eukaryotes consume smaller phytoplankton, bacteria, and archaea, and are themselves important prey for larger mesozooplankton, serving as important trophic links to higher trophic levels (Sherr and Sherr, 1994, 2002). In addition, mixotrophic microbial eukaryotes, combining the photosynthesis and the ingestion of living prey, may enhance primary production and biomass transfer to higher trophic levels (Ward and Follows, 2016; Stoecker et al., 2017). Understanding the nutritional strategies of microbial eukaryotes is essential for characterizing their ecological interactions and responses to environmental conditions.

The ocean water column is divided into five depth zones: epipelagic (0–200 m), mesopelagic (200–1,000 m), bathypelagic (1,000–4,000 m), abyssopelagic (4,000–6,000 m), and hadopelagic/hadal (>6,000 m) zones (Arístegui et al., 2009; Liu et al., 2018). The hadal zone (exclusively comprised of trenches) constitutes the deepest 45% of the vertical depth of the global ocean (Jamieson et al., 2010; Jamieson, 2015) and was known to host active and diverse biological communities (Jamieson et al., 2010; Nunoura et al., 2015; Xu et al., 2018; Liu et al., 2019b), indicating the hadal ecosystem is crucial to ocean food web. Previous surveys on the distribution of microbial eukaryotes along the water column on a global scale (Giner et al., 2020) and in specific oceanic regions (Countway et al., 2007; Not et al., 2007; Brown et al., 2009; Hu et al., 2016; Xu et al., 2017) have revealed unexpected high phylogenetic diversity of microbial eukaryotes in deep seas and a clear differentiation between surface photic and deep aphotic ocean communities. However, the maximum depth of these studies was limited to the bathypelagic zone. Jing and Xu recently conducted the first survey of microbial eukaryotes dwelling in the hadal waters (depth up to 8,727 m) of the Mariana Trench (Jing et al., 2018; Xu et al., 2018). They found significant shifts in the community composition of microbial eukaryote with depth. For instance, Alveolata dominated the surface waters whereas Stramenopiles were enriched in the deep waters. Despite these information, little is known about the microbial eukaryotic communities and metabolic potentials in the deeper seawaters (8,727–11,000 m) of the Mariana Trench, where unique microbial populations (e.g., increased amount of SAR406, hydrocarbon-degrading microbes) have been observed (Ichino et al., 2015; Nunoura et al., 2015; Liu et al., 2018, 2019b; Gao et al., 2019). In addition, whether microbial eukaryotes exhibit unique high-pressure adaptation mechanisms as found in bacteria and archaea in our previous studies (Zheng et al., 2020; Zhong et al., 2020) is still unknown. Thus, knowledge of microbial eukaryotes at the bottom of the Mariana Trench, the deepest ocean part on Earth, is necessary for a better understanding of their ecological roles and potential adaptability.

Recently, with technology advances and costs fall, metagenome has shown good performance in resolving microbial eukaryotic diversity from environmental samples (Pernice et al., 2015b; Boeuf et al., 2019; Obiol et al., 2020) free of the potential biases of PCR-dependent methods. However, there are few studies of microbial eukaryotic metabolism using metagenome due to some technical limitations like identifying eukaryotic genes (Levy Karin et al., 2020). Ocean is known to harbor a great number of eukaryotic genes especially in those unexplored zones like hadal zones, half of which however have low similarity with known genes (Carradec et al., 2018). A recent technical progress has made it possible for using metagenome to study *in situ* community-wide metabolic characteristics of microbial eukaryotes (Levy Karin et al., 2020), which will greatly expand the eukaryotic reference databases. Therefore, it is of great potential for using metagenome to study microbial eukaryotic ecology.

In this study, we extended the information of marine microbial eukaryotes to the bottom waters of the Mariana Trench by analyzing samples at seven water depths ranging from 0 to 10,500 m, a wide span from the surface to near the bottom (∼11,000 m). Metagenome derived ribosomal small subunit (SSU) gene reads were used to reveal the vertical distribution of microbial eukaryotic communities and explore the co-occurrence relationships among different clades of bacteria and microbial eukaryotes. Putative eukaryotic protein-coding genes were used to explore their metabolic potentials across the full water column. As a result, our study showed clear depth-related distribution and metabolic differences of microbial eukaryotes along the water column in the Mariana Trench.

## Materials and Methods

### Sample Collection, DNA Extraction, and Metagenomic Sequencing

The data used in this study were based on two published datasets from our previous studies (Liu et al., 2019b; Zhong et al., 2020). Those two datasets were from seawater samples of the Challenger Deep at the site 2016 (11.20°N, 142.19°E) and the site 2017 (11.38°N, 142.41°E), respectively (Table 1 and Supplementary Figure 1). The seawater samples were collected by Niskin bottles (36–48 L seawaters per depth) and then were serially filtered through 3- and 0.22-μm polycarbonate membranes. All filters were stored in liquid nitrogen onboard and at −80°C in our laboratory for DNA extraction. Detailed steps for DNA extraction, metagenomic sequencing, and quality control of reads have been described before (Liu et al., 2019b; Zhong et al., 2020). We finally obtained 17 samples from seven different water depths (surface zone: 0 m; bathypelagic zone: 2,000 and 4,000 m; hadal zone: 8,000, 9,600, 10,400, and 10,500 m) as listed in Table 1. Each sample was named according to its fraction size (0.2–3 μm, F; > 3 μm, P), sampling depth and sampling site. For example, P0_2016 represents the >3-μm sample isolated from 0 m at the site 2016. Note that samples from 2,000 and 8,000 m both have two replications and several 0.2–3-μm samples were discarded due to insufficient quantity of DNA for metagenomic sequencing. Water physicochemical parameters from March 2017 cruise were plotted in Supplementary Figure 2.

**TABLE 1:** Contexual data of 17 seawater samples from seven depths of the Mariana Trench and diversity index of microbial eukaryotes of each sample.

Sample	Site	Size (μm)	Depth (m)	Layer	18s rRNA Reads	OTUs	Richness (observed OTUs)	Shannon	Eukaryotic contigs	Eukaryotic genes
F0_2016	2016	0.2–3	0	Surface	1,581	156	118	3.49	286	1,444
P0_2016	2016	>3	0	Surface	6,679	205	71	2.49	134	494
F0_2017	2017	0.2–3	0	Surface	1,506	119	94	3.10	412	1,837
P0_2017	2017	>3	0	Surface	6,951	188	69	2.54	1,065	1,446
F20.1_2017	2017	0.2–3	2,000	Bathypelagic	1,052	112	99	3.29	268	1,236
F20.2_2017	2017	0.2–3	2,000	Bathypelagic	709	94	94	3.31	309	1,217
F40_2016	2016	0.2–3	4,000	Bathypelagic	2,268	137	76	3.06	551	1,448
P40_2016	2016	>3	4,000	Bathypelagic	4,635	167	77	3.12	6,201	16,127
F40_2017	2017	0.2–3	4,000	Bathypelagic	6,638	97	28	1.82	6,705	13,416
P40_2017	2017	>3	4,000	Bathypelagic	13,483	120	47	2.37	10,947	19,970
F80.1_2017	2017	0.2–3	8,000	Hadal	1,529	70	49	2.49	1,732	3,590
F80.2_2017	2017	0.2–3	8,000	Hadal	1,370	75	62	2.87	931	2,209
F96_2016	2016	0.2–3	9,600	Hadal	2,720	109	52	2.32	3,952	6,846
F104_2016	2016	0.2–3	10,400	Hadal	2,469	77	43	2.25	2,516	4,254
P104_2016	2016	>3	10,400	Hadal	8,747	112	40	1.50	2,363	9,981
F105_2016	2016	0.2–3	10,500	Hadal	1,254	73	56	2.47	285	1,164
P105_2016	2016	>3	10,500	Hadal	5,096	140	61	2.27	4,749	11,851
Total					68,687	490			43,406	98,530*

*98,530 genes from all the samples were finally clustered into 70,291 non-redundant genes.

### Taxonomic Profile Based on Extracted 18S rRNA Gene Reads

After quality control, clean reads ranging in size from 11.85 to 16.54 Gb were obtained from each sample (Supplementary Table 1). Metaxa2 version 2.2 (Bengtsson-Palme et al., 2015) was used to extract 18S rRNA gene reads from metagenomic data and then gave them taxonomic information based on SSU sequences in the SILVA database release 128 (Quast et al., 2012) with the default settings (reliability score cutoff = 80). Finally, a raw table of 490 operational taxonomic units (OTUs) was generated by using Metaxa2 (reads belonging to the same species were considered as an OTU) (Supplementary Data 1). As previous studies (Xu et al., 2017; Pasulka et al., 2019; Giner et al., 2020), Metazoa were not included in our study because the high number of 18S rDNA copies in the Metazoan cells may distort the relative abundance of different microbial eukaryotes. All community compositions of microbial eukaryotes in our study were based on the raw OTU table.

### Network Analysis

Intra- and inter-domain (bacteria and microbial eukaryotes) co-occurrence networks were constructed using the *igraph*, *Hmisc*, and *qvalue* libraries in R. The bacterial OTU table (Supplementary Data 2) was obtained using the Metaxa2 version 2.2 (Bengtsson-Palme et al., 2015). For both bacteria and microbial eukaryotes, only OTUs with a total proportion above 0.3% in all samples and occurring in more than three samples were retained. The pairwise Spearman’s correlations between OTUs were calculated, with a correlation coefficient > |0.7| and a *p* < 0.01 (Benjamini and Hochberg adjusted) being considered as a valid relationship. The network was visualized in Gephi.^1^

### Functional Profile Based on Putative Eukaryotic Genes

Clean reads of each sample were assembled independently using the MegaHit version 1.1.2 (Li et al., 2016). Assembled contigs of at least 1 kbp in length were used to identify potential eukaryotic contigs by EukRep with the default settings (West et al., 2018). A total of 43,406 eukaryotic contigs were obtained (Supplementary Data 3). Then, potential eukaryotic contigs were used to discover eukaryotic protein-coding genes by the new developed MetaEuk (Levy Karin et al., 2020) based on a provided database composed of three datasets: Uniclust50 (Mirdita et al., 2017), marine eukaryotic metatranscriptomes (MERC) (Steinegger et al., 2019), and Marine Microbial Eukaryotic Transcriptome Sequencing Project (MMETSP; Keeling et al., 2014; Johnson et al., 2019). This is a comprehensive and largest database focused on eukaryotes to date, and could be a valuable resource for discovering protein-coding genes from environmental samples. Note that some of predictions generated with this method are partial, because some protein-coding genes span more than one contig. Subsequently, taxonomic annotation of predicted eukaryotic genes was also performed with MetaEuk against an integrated taxonomic database composed of Uniclust90 dataset (Mirdita et al., 2017) and MMETSP (Keeling et al., 2014; Johnson et al., 2019) dataset. In this step, non-eukaryotic genes were still detected and were then deleted.

Nucleotide sequences of all predicted eukaryotic protein-coding genes from all samples were clustered at 97% similarity to generate non-redundant gene set using CD-Hit (Fu et al., 2012). Then, estimated non-redundant gene abundances (CPM – copies per million reads) based on paired end reads were determined using the Salmon version 1.2.1 (Patro et al., 2017). The Kyoto Encyclopedia of Genes and Genomes (KEGG) annotation for each gene was done using the eggNOG-mapper (Huerta-Cepas et al., 2015) with an e-value cutoff of 1e–5 against the eggNOG database. Referring to a previous study, several pathways/enzymes (Supplementary Table 2) were chosen to represent the core biogeochemical functions relevant to known metabolisms (Hu et al., 2018). Genes that appeared in multiple KEGG pathways were counted multiple times. Abundance, KEGG assignment, and taxonomic assignment of a total of 70,291 non-redundant genes were summarized in Supplementary Data 4. To understand the carbohydrate metabolism in different layers, the dbCAN2 web server (Zhang et al., 2018) was used for identifying carbohydrate-active genes with three methods: HMMER, DIAMOND, and Hotpep. Only the carbohydrate-active genes identified by two or three methods were retained.

### Statistical Analyses and Graphic Plotting

Both alpha and beta diversity analyses were performed with the *Vegan* package (Oksanen et al., 2016) in R. To equalize sequencing depth, each sample was rarefied to 709 reads (the lowest sequence number across all samples) and a total of 314 OTUs were reserved (Supplementary Data 5). Richness (observed OTUs) and Shannon indices were calculated based on rarified OTU table and the differences between different predefined groups were tested with the Wilcoxon test. For beta diversity, the non-metric multidimensional scaling analysis (NMDS) and permutational multivariate analysis of variance (ANOSIM) were performed based on Bray-Curtis dissimilarities. The heatmap was generated by the TBtools software v0.6662 (Chen et al., 2020). The upset plots and the ternary plot were generated by the UpSetR (Conway et al., 2017) and ggtern package (Hamilton and Ferry, 2018) in R, respectively.

### Access to Data

Metagenomic data of two published datasets are available in NCBI under BioProject numbers of PRJNA412741 and PRJNA541485, respectively. The nucleotide acid and amino acid sequences of each predicted eukaryotic non-redundant gene are available at zenodo (DOI: 10.5281/zenodo.3971257).

## Results

### Environmental Conditions Along the Water Column

Salinity was constant throughout the water column (34.0−34.8), while temperature and pH generally decreased from the surface (29°C; 8.2) to the trench bottom (∼1°C; 7.8) (Supplementary Figure 2). The temperature and salinity profiles were similar to previous observations (Taira et al., 2005; Nunoura et al., 2015; Xu et al., 2018). The concentration of dissolved oxygen (DO) at the sea surface was 193.6 μM and decreased to 84.9 μM at 1,000 m. Then, DO increased to 157.7 μM at 4,000 m and presented at relatively constant concentrations between 156.0 and 174.8 μM below 4,000 m. Uniform concentrations of ammonia (17.5–26.7 nM) and nitrite (0.01−0.11 μM) were observed over the depth. Silicate, phosphate, and NO_*x*_^–^ concentrations increased from the surface to 2,000 m and then remained stable at depths greater than 2,000 m.

### Alpha and Beta Diversity of Microbial Eukaryotes

**FIGURE 1:**
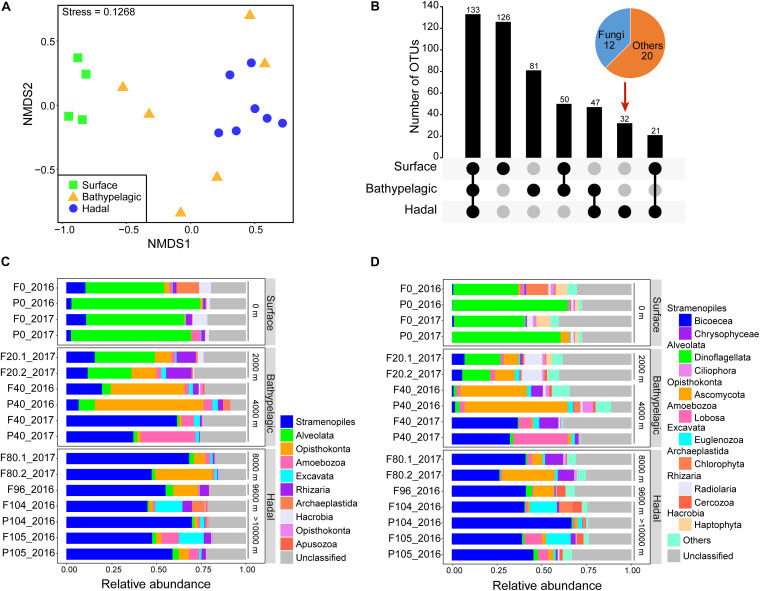
Community characteristics of microbial eukaryotes in the Mariana Trench. **(A)** Comparison of community structure based on a non-metric multidimensional scaling analysis (NMDS). Samples were grouped according to three layers: surface, bathypelagic, and hadal layers. **(B)** Core and differential operational taxonomic units (OTUs) among the surface, bathypelagic, and hadal layers. Layers included in a set were represented by filled dots in the matrix below each bar. Community composition of microbial eukaryotes from the surface to the bottom of the Mariana Trench at superphylum **(C)** and phylum/class levels **(D)**.

The diversity (richness and Shannon) of microbial eukaryotes generally decreased with depth, with a significant difference (Wilcoxon test; *p* < 0.05) between surface and hadal waters (Table 1 and Supplementary Figures 3A,B). The NMDS analysis revealed significant dissimilarity of microbial eukaryotic communities between surface and deep (bathypelagic and/or hadal) communities (Figure 1A and Supplementary Table 3; ANOSIM: *R*^2^ > 0.4, *p* < 0.05). This OTU-based grouping pattern was similar to that based on the abundance profile of functional genes (Figure 4A and Supplementary Table 4; ANOSIM: *R*^2^ > 0.5, *p* < 0.01), indicating taxonomy-related metabolic differentiations among different depths.

### Taxonomic Distribution Along the Water Column

Of the total 490 OTUs, 133 were shared among the surface, bathypelagic, and hadal layers. The surface layer had the most unique OTUs while the hadal layer possessed the least unique OTUs (mainly fungi) (Figure 1B). Throughout the water column, Stramenopiles, Alveolata, and Opisthokonta were the top three superphyla (Figure 1C and Supplementary Data 1), in consistent with the taxonomic results of functional genes (Supplementary Figures 4A,B). On average, about 20% of eukaryotic OTUs and 56% eukaryotic genes were unclassified in all samples (Figure 1C and Supplementary Figure 4B). Distinct microbial eukaryotic communities were observed at different water depths of the Mariana Trench. Alveolata represented by Dinoflagellata (36.2–64.2%) dominated the surface water, while Stramenopiles represented by Bicoecea (26.5–66.6%) dominated the deeper waters (≥4,000 m). The relative abundance of Opisthokonta (mainly Ascomycota) was higher at depths ranging from 2,000 to 9,600 m, especially at 4,000 m. The most abundant genus across all samples was *Caecitellus* (belonging to Bicoecea), comprising 18.3–58.8% of the hadal community (Supplementary Figure 5).

**FIGURE 2:**
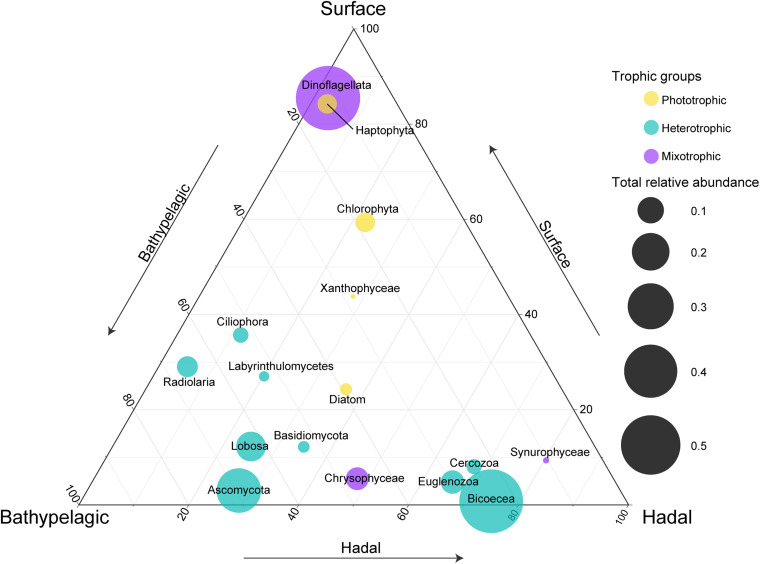
Comparisons of relative abundances for each major taxonomic group at the surface, bathypelagic, and hadal layers. Circle placement represents the relative size of relative abundances among the three layers (one layer per axis, anticlockwise with increasing relative size). Circle size represents the sum of relative abundances for each taxonomic group at three layers. Note that the taxonomic groups were listed according to their main trophic modes (mixotrophic, heterotrophic, or phototrophic).

To reveal the distribution of major taxonomic groups with different trophic modes, we compared their relative abundances at different layers (Figure 2). As expected, most phototrophic/mixotrophic (e.g., Dinoflagellata, Haptophyta, Chlorophyta) phytoplankton showed higher relative abundances at the surface, except for phototrophic Diatom and mixotrophic Chrysophyceae and Synurophyceae, which displayed higher relative abundances at the bathypelagic and/or hadal zones. All heterotrophic groups were enriched in the deep sea. Among these heterotrophic groups, Bicoecea, Euglenozoa, and Cercozoa showed the highest relative abundances at the hadal zone, indicating their potential ecological roles in the hadal trench.

### Co-occurrence Networks Between Microbial Eukaryotes and Bacteria

**FIGURE 3:**
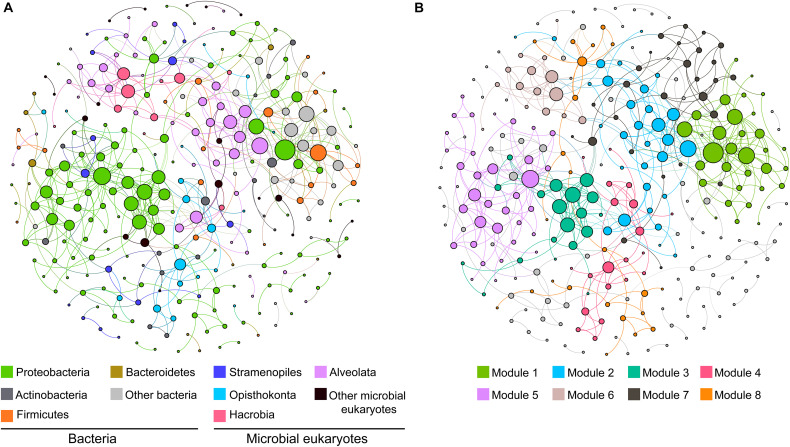
Co-occurrence networks of the dominant clades of microbial eukaryotes and bacteria based on pairwise Spearman’s correlations between OTUs. Only connection with a correlation coefficient > | 0.7| and a *p* < 0.01 was shown. The size of each node is proportional to the number of connections. The two panels show the network of all samples with OTUs colored by taxonomy **(A)** and modularity **(B)**.

To explore the ecological linkages among microbial eukaryotes and between microbial eukaryotes and bacteria, a correlation-based network analysis was conducted (Figures 3A,B). The network was composed of 291 nodes (101 eukaryotic OTUs and 190 bacteria OTUs) and 558 edges (correlations). Correlations within eukaryotic taxa were stronger than those between bacteria and eukaryotes (Figure 3A). Modularity analysis revealed eight major modules (subunits with highly inter-connected nodes) (Figure 3B). Most of these modules were comprised of a group of OTUs that were phylogenetically close or even belonged to the same clade. For example, module 3 and 5 were predominated by Proteobacteria OTUs and module 2 was dominated by Alveolata OTUs (Figures 3A,B). Thus, taxonomic relatedness plays a key role in determining the network modular structure. Clades with similar trophic modes also tended to co-occur. For example, module 6 consisted of Alveolata (mainly phototrophic/mixotrophic Dinoflagellata) and Hacrobia (mainly phototrophic Haptophyte) OTUs, and module 4 comprised metatrophic Opisthokonta (totally fungi) and Actinobacteria OTUs. Additionally, connections between ecologically linked clades were observed. For example, Stramenopiles (mostly flagellates) OTUs were dispersedly distributed in the network and connected with some bacterial phyla.

### Community-Wide Metabolic Potentials Across the Whole Water Column of the Mariana Trench

**FIGURE 4:**
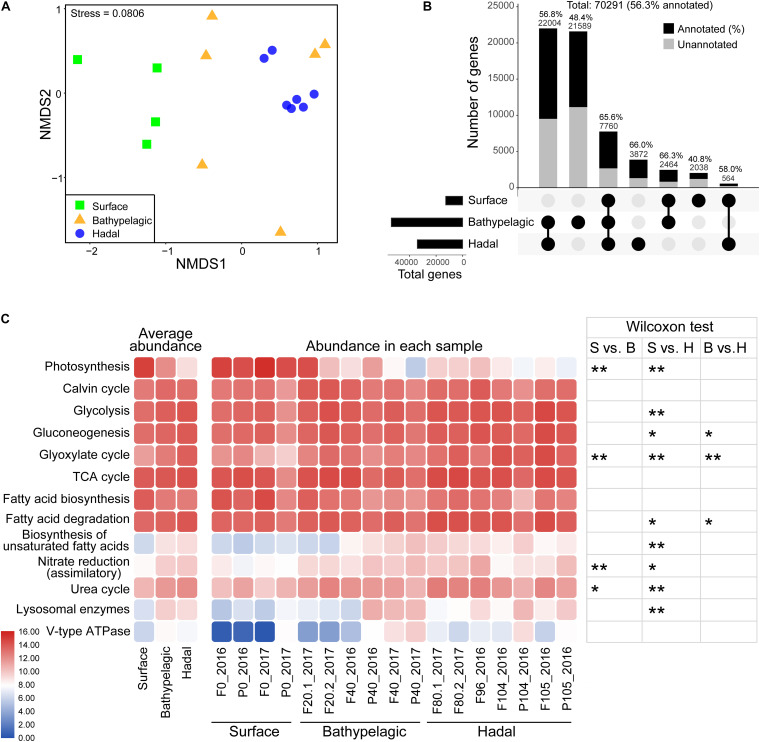
Metabolic characteristics of microbial eukaryotes in the Mariana Trench. **(A)** Differential analysis of functional structure of microbial eukaryotes based on a NMDS analysis. Samples were grouped according to three layers: surface, bathypelagic, and hadal layers. **(B)** Shared and unique non-redundant genes between epipelagic, bathypelagic, and hadal zones. Layers included in a set were represented by filled dots in the matrix below each bar. The number of genes that had KEGG identities was represented by black (also given as percentages). **(C)** Comparisons of key pathways at different samples. Heatmaps show the average abundances (CPM: copies per million reads) of KEGG pathways in the epipelagic, bathypelagic, and hadal zones (left) and their abundances in all the samples (medium). Abundance values generating this heatmap were log2 scaled. Wilcoxon test was used for significance test (right); *p < 0.05, **p < 0.01. S, surface; B, bathypelagic; H, hadal.

Of the total 70,291 eukaryotic non-redundant genes (56.3% had KEGG identities), most were from the bathypelagic and hadal waters (Figure 4B). Only 7,760 genes were shared within the surface, bathypelagic, and hadal layers, mainly belonging to Stramenopiles and Opisthokonta (Figure 4B and Supplementary Figure 6). The bathypelagic and hadal layers had the most common genes (22,004, 56.8% had KEGG identities). Bathypelagic layer had the most unique genes, over 70% of which were taxonomically unclassified. Compared to the surface (40.6%), the hadal zone had a higher percentage of genes (66%) that can be assigned to KEGG identities. There were 3,872 genes unique for the hadal layer, mainly belonging to Stramenopiles (Figure 4B and Supplementary Figure 6). Among the top five abundant hadal-unique genes, two were involved in DNA formation and stabilization [K03163 (*top1*; DNA topoisomerase I) and K03530 (*hupB*; DNA-binding protein HU-beta)], and three were related to alcohol dehydrogenation [K13979 (*yahK*; uncharacterized zinc-type alcohol dehydrogenase-like protein), K00002 (*adh*; alcohol dehydrogenase), and K13953 (*adhP*; alcohol dehydrogenase, propanol-preferring)] (Supplementary Figure 7 and Data 6).

Most pathways/enzymes had significant differences (*p* < 0.05) in abundance between the surface and the hadal zones (Figure 4C and Supplementary Tables 5, 6), which may be due to the dramatically different environments. Genes associated with biosynthetic pathways like photosynthesis (*p* < 0.01) and fatty acid biosynthesis were higher in abundance at the surface compared to deeper depths, except for the Calvin cycle which showed similar abundance among all depths. Genes involved in several carbon metabolism pathways (Glycolysis, Gluconeogenesis, Glyoxylate cycle, fatty acid degradation, and biosynthesis of unsaturated fatty acid), nitrogen metabolism pathways (assimilatory nitrate reduction and urea cycle), and digestion (lysosomal enzymes and V-type ATPase) were higher in abundance in the deep sea (bathypelagic and/or hadal zones).

**FIGURE 5:**
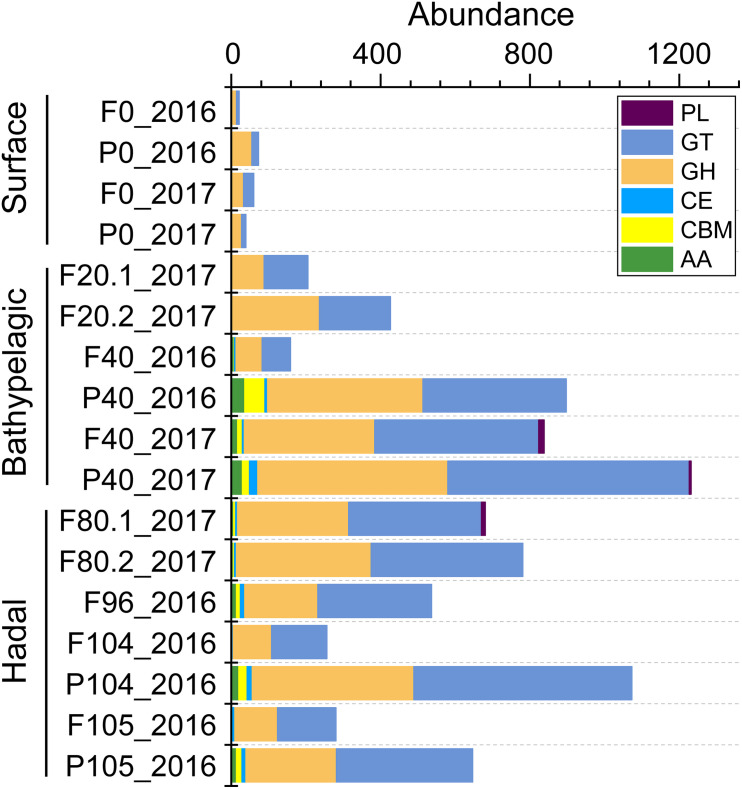
The abundance (CPM) of each enzyme family involved in carbohydrate metabolism across all the depths. AAs, auxiliary activities; CBMs, carbohydrate-binding modules; CEs, carbohydrate esterases; GHs, glycoside hydrolases; GTs, glycosyltransferases; PLs, polysaccharide lyases.

To understand carbohydrate metabolism characteristics in different layers, genes were assigned to different enzyme families and only 309 (0.44% of all 70,291 genes) genes were annotated (Supplementary Table 7). The total abundance of genes involved in six enzyme families was higher in the deep sea (Figure 5). The main enzyme families increasing in the deep seawater (≥2,000 m) were glycosyltransferases (GTs, catalyzing the formation of the glycosidic linkage to form a glycoside) and glycoside hydrolases (GHs, catalyzing the hydrolysis of O–, N–, and S–linked glycosides), which are crucial to carbohydrate biosynthesis and degradation, respectively.

## Discussion

### Stratified Community Structure of Microbial Eukaryotes

The vertical changes in community composition and metabolic potentials of microbial eukaryotes across the full water column of the Mariana Trench (0–10,500 m) were investigated through metagenomic sequencing in this study. Similar to previous reports in the Mariana Trench (Jing et al., 2018; Xu et al., 2018) and other oceanic regions (Countway et al., 2007; Brown et al., 2009; Xu et al., 2017; Giner et al., 2020), decreased diversity with depth and depth-related community separation were observed. Abiotic factors like depth and depth-related environmental conditions (e.g., temperature, hydrostatic pressure, nutrient availability) have been recognized as the important factors in shaping the distribution of microbial eukaryotic communities on a regional scale as seen in the above references.

Other than abiotic factors, complex microbial interactions (e.g., host–parasite, predator–prey, symbiotic relationships) were also crucial for community assembly (Steele et al., 2011; Faust and Raes, 2012; Lima-Mendez et al., 2015) and may be partially revealed by co-occurrence networks (Faust and Raes, 2012; Liu et al., 2019a). Our results showed that interactions mainly occurred among phylogenetically close taxa, which might due to similar lifestyles and metabolic or physiological characteristics. Consistent with a previous study (Gilbert et al., 2012), associations within domains (bacteria or microbial eukaryotes) were stronger than those between them. Despite this, several connections between bacteria and microbial eukaryotes were observed, indicating their potential ecological linkages. For example, the most connected eukaryotic clade with bacteria was Stramenopiles (Figure 3), which was likely due to their role as important predators of prokaryotes (Burki, 2014). Here, we only discussed the interactions between microbial eukaryotes and bacteria. Interactions between microbial eukaryotes, and other taxa like bigger megafauna [e.g., amphipods and snailfish; (Jamieson et al., 2010)], viruses and archaea may be also important for community assembly of microbial eukaryotes and still need further study.

### Enriched Groups at the Bathypelagic and/or Hadal Zones

As expected, the surface waters were dominated by phototrophic/mixotrophic phytoplankton accompanied by a high abundance of genes involved in biosynthesis (photosynthesis and fatty acid synthesis) (Figures 2, 4C), which was also observed in a metatranscriptomic study at the San Pedro Ocean (Hu et al., 2018). Specifically, the relative abundances of phototrophic Diatom and mixotrophic Chrysophyceae were higher in the bathypelagic and hadal zones of the Mariana Trench (Figure 2). Indeed, the presence of living phytoplankton like diatoms, cyanobacteria, and dinoflagellates in the dark ocean has been reported, and fast-sinking or changing nutritional strategies may be the possible explanations (Jiao et al., 2014; Agusti et al., 2015; Xu et al., 2017; Giner et al., 2020). As for Chrysophyceae, they are common bacteriovores and were most abundant phytoplankton frequently detected in the water column at depths of 3,000–4,000 m worldwide (Pernice et al., 2015b).

Fungi perform vital functions as decomposers, driving nutrient cycles in detritus environments, and as parasites and symbionts in the ecosystems (Webster and Weber, 2007). Similar to previous studies (Xu et al., 2018; Wang et al., 2019), the relative abundance of fungi (mainly branched with Ascomycete) was highest in the bathypelagic zone (Figure 2). In addition, a fungi-like group labyrinthulomycetes was also enriched in the bathypelagic zone (Figure 2). Marine snow, aggregation of organisms, and organic matter like fecal pellets and phytodetritus, is important to the biological pump, transporting particulate carbon to greater depths (Turner, 2015). Fungi and labyrinthulomycetes have been reported to be the dominant eukaryotes of bathypelagic marine snow and have the potential to significantly contribute to the degradation of organic matter in the deep sea (Bochdansky et al., 2016). In addition, fungi prefer utilizing organic matter with high C:N ratio (Raghukumar, 2012), which was reported to increase with depth (Hopkinson and Vallino, 2005). Therefore, these saprotrophic organisms have the potential to significantly complement bacteria and archaea in utilizing and recycling of nutrients in deep seas.

Our study complemented the information of microbial eukaryotes in the bottom waters (≥9,600 m) of the Mariana Trench compared to previous studies (Jing et al., 2018; Xu et al., 2018). We found Bicoecea, Euglenozoa, and Lobosa were the dominant heterotrophic protists in the bottom waters (Figure 1D), indicating their potential high-pressure adaptability. Moreover, Bicoecea was the most dominant group in the shallower hadal zone, which was only found in one of two previous studies (Jing et al., 2018; Xu et al., 2018). Considering different primers applied to recover microbial eukaryotes in their studies, primer bias may be a main factor resulting in this differentiation (Stoeck et al., 2006). In addition, significant increase in relative abundances of Bicoecea along the water column was also reported on a global scale (Giner et al., 2020). The dominant genus of Bicoecea is *Caecitellus* (Supplementary Figure 5), which is a group of small flagellates exerting high grazing activity on prokaryotes and other eukaryotes (Boenigk and Arndt, 2002) and showing general tolerance to high pressure (up to 550 bar under experimental conditions) (Atkins et al., 1998; Živaljić et al., 2018), indicating their important role in deep seas. Small heterotrophic protists are considered as the first consumers of prokaryotic biomass and main consumers of suspended organic matter, promoting organic matter remineralization (Boenigk and Arndt, 2002). Recent studies showed higher-than-expected grazing activity by protists down to mesopelagic depths (Pernice et al., 2015a). Abundant heterotrophic bacteria like SAR406 and hydrocarbon-degrading microbes have been reported in the hadal zone of the Mariana Trench (Nunoura et al., 2015; Gao et al., 2019; Liu et al., 2019b), probably breeding microbial eukaryotes therein. Thus, heterotrophic protists may have capabilities of regenerating nutrients and inorganic molecules in the hadal zone.

### Metabolic Changes in the Hadal Zone Compared to the Surface

In the hadal zone, we observed high abundances of genes involved in digestion (lysosomal enzymes and V-type ATPase) and carbohydrate metabolism (glycosyltransferases and glycoside hydrolases) (Figures 2, 4C), all of which are important in the heterotrophic process. For example, lysosomal enzymes are involved in many cellular processes like the internal breakdown and recycling of biomolecules (Eskelinen and Saftig, 2009; Settembre et al., 2013) and are highly expressed in heterotrophic flagellates (Labarre et al., 2020). V-type ATPases play a role in digestive processes by lowering the pH in phagosomes (Finbow and Harrison, 1997). In view of abundant heterotrophic clades found in the hadal zone, it is reasonable that genes associated with heterotrophic processes were enriched therein.

High hydrostatic pressures reduce the fluidity of lipid bilayers and the reversibility of their phase transitions, ultimately leading to the denaturation and functional disorder of membrane-associated proteins (Chong et al., 1983; Kato et al., 2002). Many heterotrophic protists have been reported to be able to survive exposure to high hydrostatic pressure, indicating their potential genetic adaptation (Živaljić et al., 2018). However, their adaptation mechanism is still unknown. In our study, we found increased abundance of genes responsible for biosynthesis of unsaturated fatty acid at depths exceeding 4,000 m (Figure 4C). Likewise, several biochemical studies have suggested that membranes of deep-sea-adapted organisms contain a higher weight percentage of unsaturated fatty acids than the equivalent membranes of shallow-sea species (Behan et al., 1992; Fang et al., 2000). It has been acknowledged that an increase in fluidity of membranes by incorporation of unsaturated fatty acids plays a role in the bacterial survival at high hydrostatic pressures (DeLong and Yayanos, 1985; Allen et al., 1999) and low temperatures (DeLong and Yayanos, 1986). Therefore, we speculated unsaturated fatty acid may be also effective in resisting high hydrostatic pressures for microbial eukaryotes through improving the fluidity of membrane.

The hadal zone had a relatively higher percentage of annotated genes than the surface zone (Figure 4B). The reason might be the higher diversity of both prokaryotes and eukaryotes in the shallow ocean compared to the deeper ocean (Nunoura et al., 2015; Jing et al., 2018; Tian et al., 2018; Xu et al., 2018), as many organisms cannot adapt to the extreme deep-sea environments. Of the five most abundant genes unique in the hadal zone, two (*Top1* and *hupB*) were involved in DNA related processes like replication and transcription (Champoux, 2001; Gupta et al., 2014). Indeed, high pressure treatment was found to affect DNA structure in bacteria (Bartlett, 2002). Thus, these two unique genes might play a role in stabilizing DNA structure in microbial eukaryotes in the hadal zone, which needs further study.

## Conclusion

This study investigated the distribution of microbial eukaryotes along the water column of the deepest known ocean. Stratification in community structure and shift of dominant taxonomies with depth were observed, indicating potential influence of depth-related environmental conditions on community composition. In addition, co-occurrence analysis showed that microbial interactions were also important for community composition. Intra-domain connectivity was higher than that between domains (bacteria and microbial eukaryotes), and clades with close phylogenetic relationships or similar trophic modes tend to coexist. Metagenome was firstly used for exploring the community-wide metabolic potentials of microbial eukaryotes in environmental samples, revealing the coordinating variations between metabolic characteristics and community composition. Metabolic changes like increased abundance of genes involved in the biosynthesis of unsaturated fatty acid, and abundant unique genes related to the formation and stabilization of DNA structure in hadal seawaters may help microbial eukaryotes adapt to high hydrostatic pressure. Our study is only a snapshot of microbial eukaryotic metabolism in the Mariana Trench. More efforts are needed to help improve our understanding of how microbial eukaryotes survive in the deep sea and respond to extreme conditions at the transcriptional level. Furthermore, this study indicated that with comprehensive databases metagenomics can be an important approach to studying microbial eukaryotic diversity and has potentials for exploring community-wide metabolic characteristics.

## Data Availability Statement

The datasets presented in this study can be found in online repositories. The names of the repository/repositories and accession number(s) can be found in the article/Supplementary Material.

## Author Contributions

X-HZ designed the experiments. X-YZ performed the experiments, analyzed the data, and wrote the draft. JL, C-XX, and JT provided great help on bioinformatics analyses and manuscript writing. All authors read and approved the final manuscript.

## Conflict of Interest

The authors declare that the research was conducted in the absence of any commercial or financial relationships that could be construed as a potential conflict of interest.

## Footnotes

1 https://gephi.org
