# Supplementary material for: Shift and Metabolic Potentials of Microbial Eukaryotic Communities Across the Full Depths of the Mariana Trench
Source: Front Microbiol. 2021 Jan 18;11:603692. doi: 10.3389/fmicb.2020.603692 (PMC7848797; doi:10.3389/fmicb.2020.603692)
Supplement: Supplementary file 7 [file Image_1.pdf]

Supplementary figures

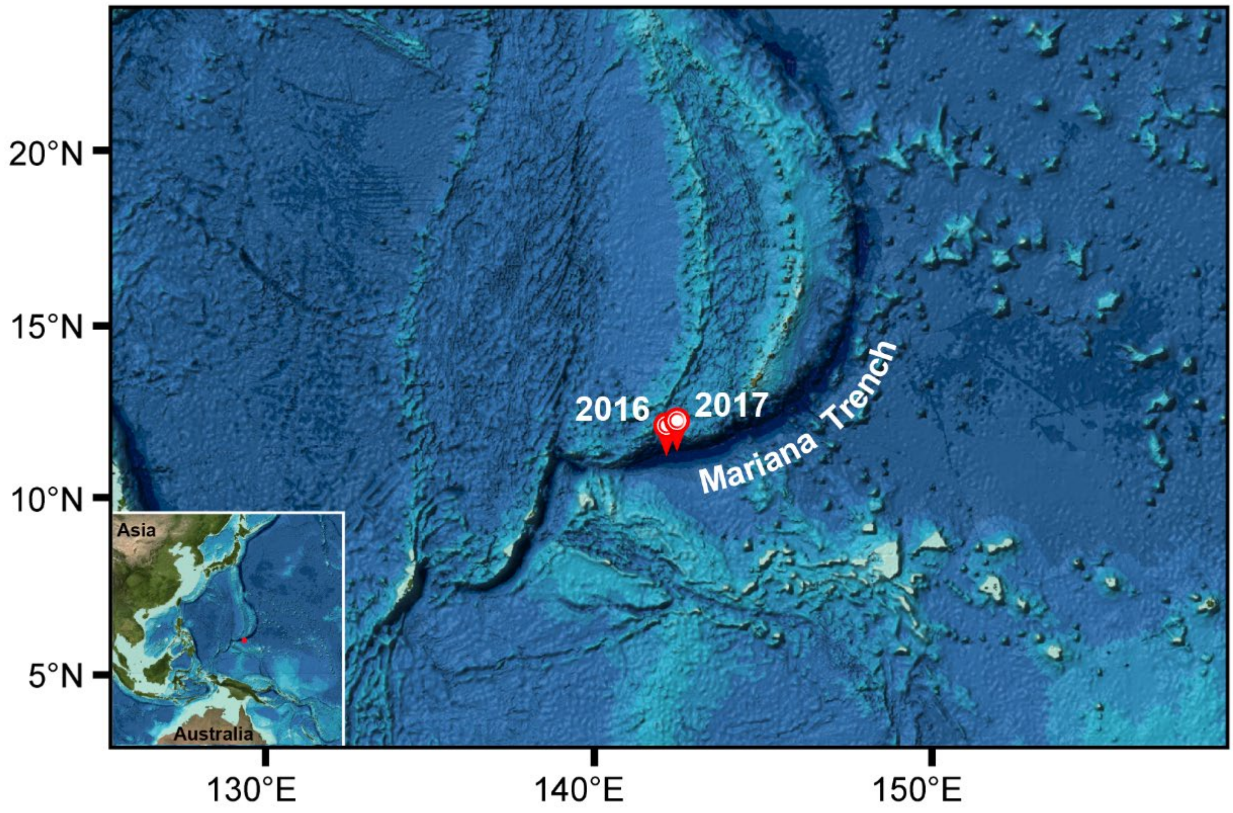

Figure S1. Sampling sites at the Challenger Deep of the Mariana Trench. The map was reproduced from the GEBCO world map 2014 (www.gebco.net).

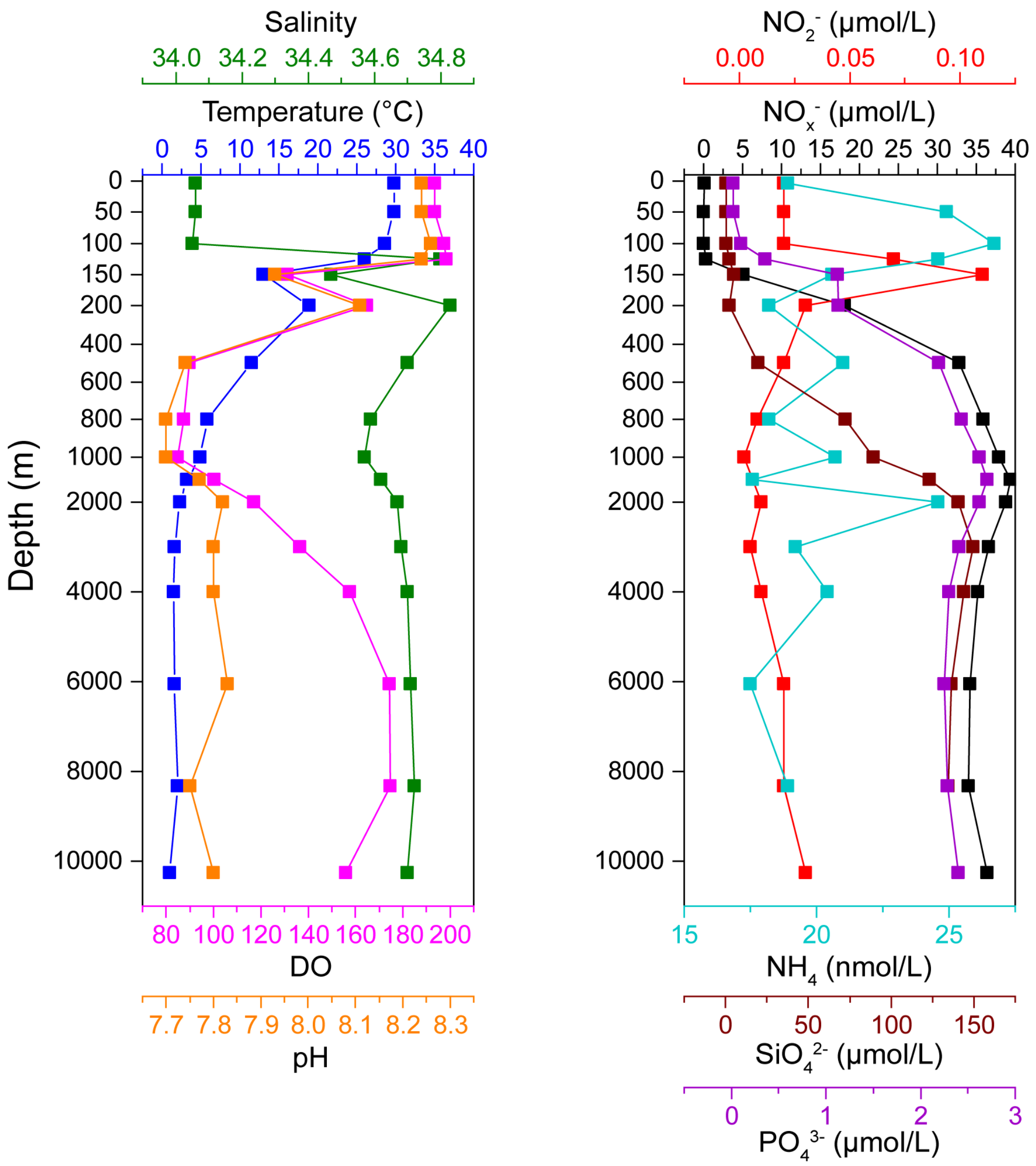

Figure S2. Environmental parameters along the water column at the Challenger Deep of the Mariana Trench. Data of 0-8,320 m were from Mar. 2017 cruise and data of 10,257 m were from a previous report (Nunoura et al., 2015).

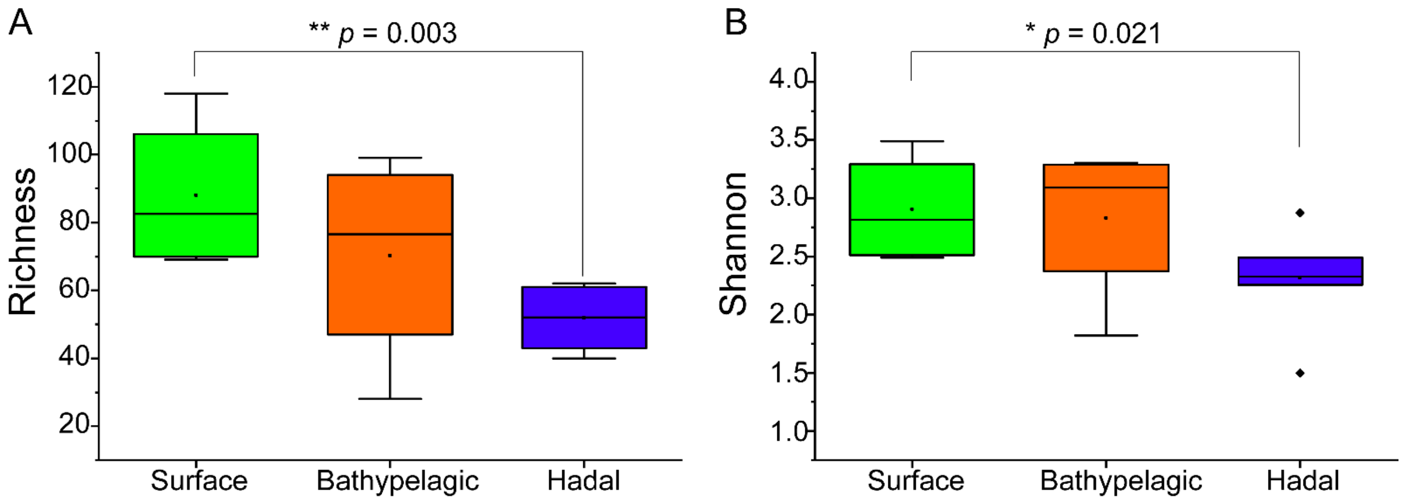

Figure S3. Comparison of richness (observed OTUs) and Shannon indices between different groups. Surface, bathypelagic and hadal groups. *$p < 0.05$, **$p < 0.01$; Wilcoxon test.

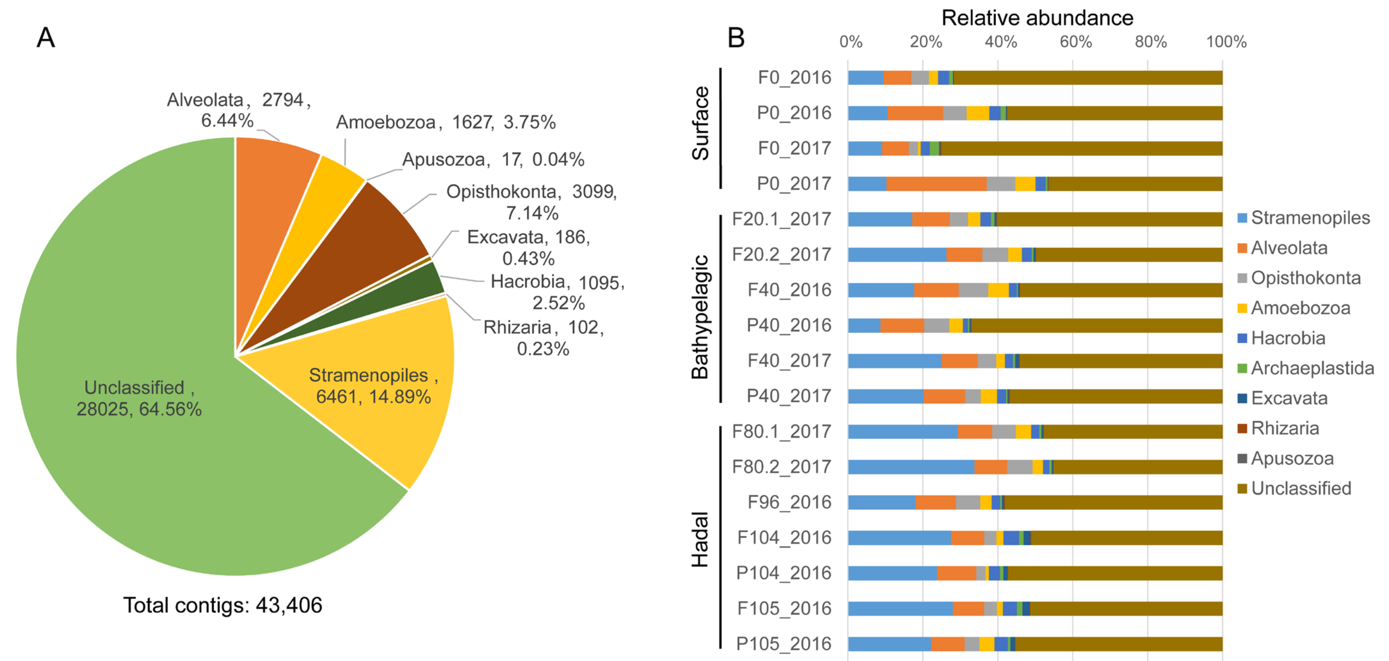

Figure S4. Taxonomic assignment of the total 43,406 eukaryotic contigs obtained from all samples **(A)**. Relative functional gene abundances of different superphyla across all samples **(B)**. The abundance of each superphylum is the total abundance (CPM: copies per million reads) of genes belonging to it. To enable comparisons of community composition based on 18S rDNA genes and functional genes, metazoan functional genes were not included in the analysis.

Genus	F0_2016	P0_2016	F0_2017	P0_2017	F20.1_2017	F20.2_2017	F40_2016	P40_2016	F40_2017	P40_2017	F80.1_2017	F80.2_2017	F96_2016	F104_2016	P104_2016	F105_2016	P105_2016
Caecitellus	0.00506	0.00105	0.00598	0.00029	0.05418	0.03385	0.00397	0.01381	0.28382	0.26389	0.30020	0.18321	0.36765	0.36006	0.58820	0.35008	0.39403
Platyamoeba	0.00000	0.00030	0.00000	0.03999	0.01616	0.01693	0.00441	0.00345	0.05182	0.26418	0.01962	0.00730	0.00074	0.00324	0.00446	0.03270	0.02767
Paraphysomonas	0.00253	0.00000	0.00133	0.00000	0.00475	0.00000	0.03395	0.00000	0.03299	0.00727	0.00458	0.00730	0.00294	0.00648	0.00057	0.01595	0.00451
Neobodo	0.00000	0.00000	0.00000	0.00029	0.00095	0.00000	0.00044	0.00000	0.00889	0.00126	0.00000	0.00073	0.00037	0.07898	0.01132	0.05901	0.00216
Aspergillus	0.00253	0.00060	0.00000	0.00000	0.00285	0.00564	0.03924	0.05933	0.00000	0.00030	0.00262	0.01095	0.00221	0.00000	0.00069	0.00000	0.00098
Syndiniales	0.02277	0.00958	0.06042	0.00834	0.03992	0.00987	0.00132	0.00043	0.00090	0.00067	0.00196	0.00000	0.00147	0.00000	0.00011	0.00080	0.00020
Gymnodiniphycid	0.00949	0.01931	0.00531	0.01525	0.00095	0.00000	0.00088	0.00043	0.00000	0.00052	0.00000	0.00000	0.00037	0.00000	0.00023	0.00000	0.00059
Rhynchomonas	0.00000	0.00000	0.00266	0.00000	0.00000	0.00282	0.00000	0.00000	0.01235	0.00326	0.01046	0.01387	0.00000	0.00486	0.00194	0.01914	0.00098
Amoebophrya	0.01265	0.00434	0.03054	0.00331	0.01901	0.02257	0.00088	0.00022	0.00060	0.00007	0.00262	0.00000	0.00037	0.00000	0.00000	0.00000	0.00000

Figure S5. The relative abundances of the top 10 abundant genera across all the samples.

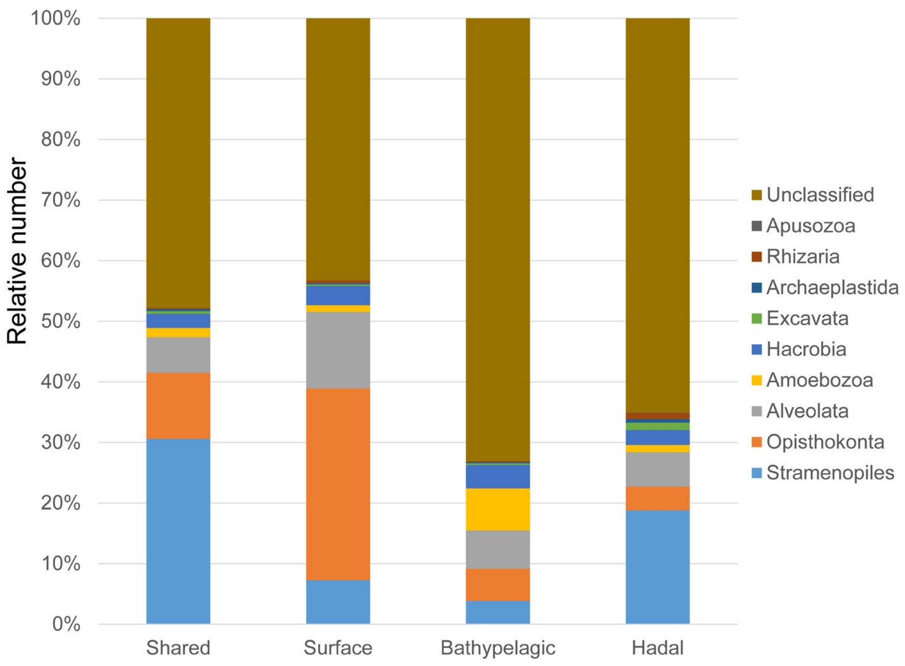

Figure S6. Taxonomic assignment for functional genes shared and unique among different layers. Abbreviation: Shared, shared genes among three layers; Surface, surface unique genes; Bathypelagic, bathypelagic unique genes; Hadal, hadal unique genes. See main text Figure 4B.

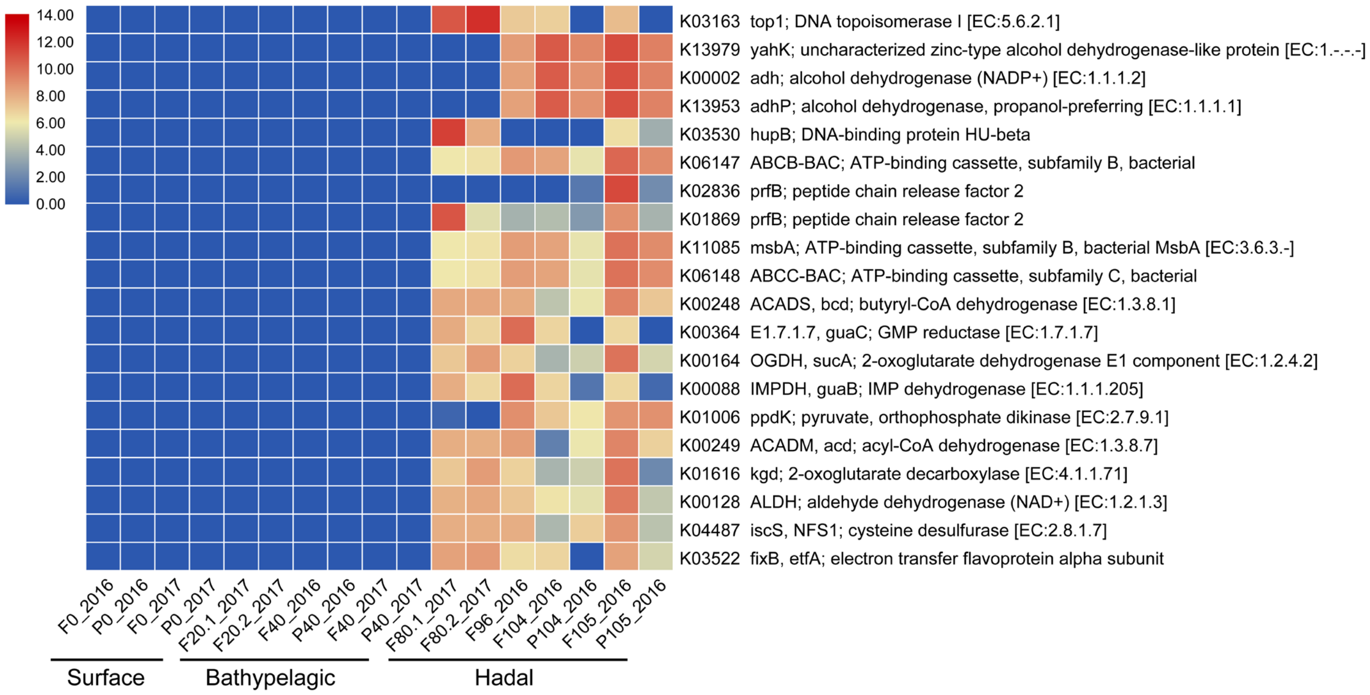

Figure S7. Abundance (CPM) of the most abundant 20 genes unique in the hadal zone. See main text Figure 4B. Abundance values generating this heatmap were log2 scaled.

References:

Nunoura, T., Takaki, Y., Hirai, M., Shimamura, S., Makabe, A., Koide, O., et al. (2015). Hadal biosphere: insight into the microbial ecosystem in the deepest ocean on Earth. *Proc. Natl. Acad. Sci. U.S.A.* 112, E1230-E1236. doi: 10.1073/pnas.1421816112
